# Nasal Mites of Kelp Gulls (*Larus dominicanus*) in Brazil: Geographic Distribution and Parasitological Indices

**DOI:** 10.1007/s11686-026-01396-w

**Published:** 2026-09-16

**Authors:** Silvia Bainy Gastal, Carolina Silveira Mascarenhas, Leandro Bugoni

**Affiliations:** 1 Laboratório de Aves Aquáticas e Tartarugas Marinhas, Instituto de Ciências Biológicas, Universidade Federal do Rio Grande - FURG, Rio Grande, Brazil; 2 Programa de Pós-Graduação em Oceanografia Biológica, Universidade Federal do Rio Grande - FURG, Rio Grande, Brazil; 3 Laboratório de Parasitologia, Área Interdisciplinar de Ciências Biomédicas, Faculdade de Medicina, Universidade Federal do Rio Grande - FURG, Rio Grande, Brazil

**Keywords:** Acarology, Infection indices, Laridae, *Larinyssus orbicularis*.

## Abstract

**Purpose:**

This study aims to investigate the occurrence, geographic distribution, and parasitological indices of nasal mites in Kelp Gulls (*Larus dominicanus*) from southern Brazil, as well as to assess whether infection indices differ according to host gender, age class and latitudinally.

**Methods:**

Nasal cavities from 171 gulls collected in southern and southeastern Brazil were inspected for the presence of mites. Recovered specimens were clarified, mounted on microscope slides, and identified using light microscopy, with selected specimens examined by scanning electron microscopy (SEM). Parasitological indices were analyzed and compared between male and female hosts, as well as among adults, juveniles, and immature gulls.

**Results:**

*Larinyssus orbicularis* was detected in 32.7% of the gulls examined, with parasitized hosts recorded in both Rio Grande do Sul and Santa Catarina states, and absent in northern areas. The prevalence of mites was similar among male and female hosts. However, juvenile gulls exhibited a significantly lower prevalence than immatures and adults. A record-high infection intensity of 197 mites was recorded from a single individual, substantially exceeding previous reports for this parasite genus. Heavy infections (> 50 mites) occurred in 21.5% of parasitized gulls.

**Conclusions:**

This study highlights the expanded geographic distribution of *L. orbicularis* and provides baseline parasitological data for Kelp Gull. The high infection intensities observed, including heavily parasitized individuals, indicate that nasal mite infections may constitute an important but largely overlooked component of host health.

## Introduction

Birds host a hyperdiverse assemblage of symbiotic animals occupying a wide range of body microhabitats. Among these organisms, mites are one of the most diverse and frequently recorded groups in association with birds, with at least 3,000 species currently described [1, 2]. Five families of acariform mites (Cloacaridae, Cytoditidae, Ereynetidae, Turbinoptidae) and parasitiform (Rhinonyssidae) have evolved independently as parasites in the nasal passages or lungs of birds [2]. Among them, rhinonyssids are obligate haematophagous endoparasites that dwell the membrane of nasal cornets, in or near the mucous lining of the nasal cavity, although they have been found in the lungs, trachea, and body cavity [3, 4].

Rhinonyssidae is distributed into eleven genera: *Larinyssus* Strandtmann, 1948, *Locustellonyssus* Bregetova, 1965, *Mesonyssus* Fain, 1960, *Ptilonyssoides* Vitzthum, 1935, *Ptilonyssus*, Berlese & Trouessart, 1889, *Rallinyssus* Strandtmann, 1948, *Rhinoecius* Cooreman, 1946, *Rhinonyssus* Trouessart, 1894, *Sternostoma* Berlese & Trouessart, 1889, *Tinaminyssus* Strandtmann & Wharton, 1958 and *Vitznyssus* Castro, 1948 [5, 6]. Some genera are found only in particular family of hosts (*e.g. Larinyssus* in Laridae birds), while others parasitize birds of different orders (*e.g. Ptilonyssus*) [7, 8].

Three rhinonyssids genera occur in seabirds and shorebirds: *Larinyssus*, *Rhinonyssus* and *Sternostoma* [8–10]. *Larinyssus* currently includes six species distributed exclusively on waders and gulls (Charadriiformes): *L. orbicularis* Strandtmann, 1948, *L. petiti* Gretillati, 1961, *L. benoiti* Fain, 1961, *L. sterna* Fain & Holland, 1972, *L. substerna* Butenko, 1975 and *L. iohanssenae* Dimov, 2013 [11–16].

Larids are the most familiar charadriiform birds of every shore and occupy a wider range of habitats [17]. While most species live in coastal areas, some are pelagic for most of the year and others live far inland, mostly near water, but also in some extremely xeric environments that are within a daily commute to water [18]. Among the Laridae, 56 consist of gull species, distributed in 11 genera, while *Larus* is the most diverse, with 26 species [19]. The Kelp Gull (*Larus dominicanus* Lichtenstein, 1823) is the most abundant species of gull in the southern hemisphere [20]. The species has a broad distribution across Africa, from Namibia to the Cape Province and Madagascar; Australia, New Zealand, Peru, Brazil, Tierra del Fuego, the Falkland Islands, Sub-Antarctic islands, and Antarctica, where populations may be resident, migratory, or dispersive [21]. In Brazil, it is distributed predominantly between Rio Grande do Sul and Espírito Santo states [22, 23].

Several species of gulls, including *L. dominicanus*, are hosts to rhinonyssid mites and these parasites are of special interest to veterinary and medical entomologists due to their hematophagous habit [10, 24]. The invasion of the airways of birds by Rhinonyssidae mites could injure the nasal epithelium and blood vessels and can cause pulmonary congestion, rhinitis and sinusitis [25–27]. Dimov [28] named the disease caused by these mites as rhinonyssidosis avium. Pathological conditions including anorexia, aphonia, and cachexia caused by *Mesonyssus*, *Sternostoma* and *Ptilonyssus* species, as well as infections causing aircystitis, tracheitis, and pneumonia, which often lead to the death of the hosts, were reported in captive birds [28, 29].

Due to the potential harm to birds reported in previous studies and the limited information available on mite infestations in gulls, this study aims to investigate the presence of nasal mites and their infection indices in the Kelp Gull. In addition, it aims to investigate whether prevalence, mean abundance and mean intensity differ among male and female hosts and among juvenile, immature, and adult age classes, as well as to characterize the geographic distribution of parasite records in Brazil.

## Materials and Methods

### Collection of Hosts

One hundred and seventy-one Kelp Gulls were collected and examined between 2015 and 2022. Samples consisted of 84 females (14 juveniles, 10 immatures and 60 adults), 78 males (14 juveniles, 20 immatures and 44 adults) and nine individuals whose gender was not determined (6 juveniles and 3 adults). Birds were classified by age as juvenile, immature or adult based on plumage, and gender was determined by visual examination of the gonads at necropsy [19, 30].

**Fig. 1:**
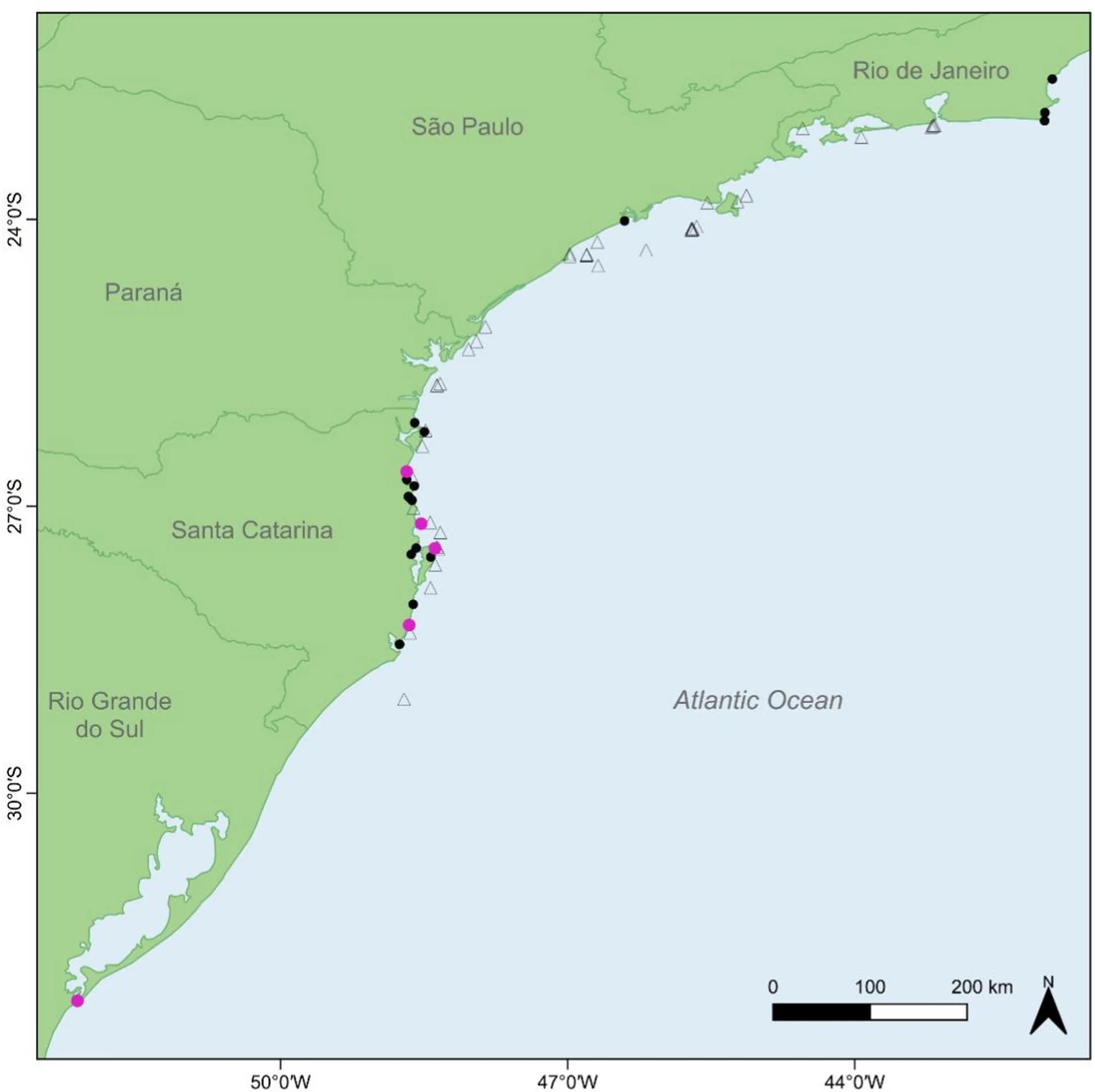
Distribution of sampling sites for Kelp Gulls (*Larus dominicanus*). Purple dots indicate locations where at least one individual was parasitized by *Larinyssus orbicularis*, while black dots represent locations with no recorded parasitized hosts. The triangles indicate the breeding areas of Kelp Gulls in Brazil

Birds were collected during routine beach surveys (*n* = 25) or after death under care in Marine Animal Recovery Centers (*n* = 146). The birds were collected in the Brazilian states of Rio Grande do Sul (municipality of Rio Grande *n* = 21), Santa Catarina (municipalities of Balneário Camboriú *n* = 1, Barra Velha *n* = 1, Bombinhas *n* = 4, Florianópolis *n* = 108, Garopaba *n* = 3, Governador Celso Ramos *n* = 2, Imbituba *n* = 3, Itajaí *n* = 1, Itapoá *n* = 1, Laguna *n* = 2, Navegantes *n* = 3, Palhoça *n* = 2, Penha *n* = 2, Piçarras *n* = 1, São Francisco do Sul *n* = 5), São Paulo (municipality of Praia Grande *n* = 1) and Rio de Janeiro (municipalities of Arraial do Cabo *n* = 3, Cabo Frio *n* = 1 and Rio das Ostras *n* = 6) (Fig. 1). All carcasses were collected within 24 h of animal's death and none of the examined birds had received antiparasitic treatment.

### Collection, Preparation and Identification of Mites

Mites were searched for in the nasal cavities of gulls, through dissection according to Gastal et al. [31], then washing in running water on a 150 μm mesh sieve. The resulting contents were inspected under a stereomicroscope, and mites were stored in 70% ethanol. For microscopic analysis, specimens were clarified in Nesbitt solution and mounted in Hoyer's medium between the slide and cover slip. Mite identification agreed with Strandtmann, Fain & Holland and Butenko [11, 14, 15]. Vouchers were deposited in the *Coleção Acarológica do Instituto Butantan* (IBSP), Brazil (IBSP, N. 28,332–28,333). Due to the high number of parasites collected, a subsample corresponding to 10% of the total number of specimens collected was mounted on slides and examined under a microscope for species identification. At least one mite from each parasitized host was prepared and analyzed on the slides.

To perform the scanning microscopy, 30 selected mites were prepared according to Dimov [32]. Photographs were taken using a Scanning Electron Microscope, in high- and low-vacuum mode, Jeol^®^, JSM—6610LV, with EDS microprobe.

### Parasitological Indices

Parasitism prevalence (P%), mean abundance (MA) and mean intensity (MI) were estimated according to Bush et al. [33]. The prevalence of mites by gender and by age class was analyzed using Pearson's chi-square test. Mean abundance and mean intensity between males and females were assessed using the nonparametric Wilcoxon (Mann-Whitney) test. For comparisons between age classes, the nonparametric Kruskal-Wallis test was used with significant results followed by Dunn's post hoc test. Bonferroni correction was applied to pairwise comparisons of prevalence and mean abundance, with significance set at *p* < 0.017 (0.05/3 comparisons). The analyses were conducted in BioEstat 5.3 software [34].

As no previous classification of *Larinyssus* infection intensity in Kelp Gulls is available, a semi-quantitative classification of infection intensity was proposed based on the distribution of mite counts observed among the birds examined in this study. Accordingly, infections were classified as low (1–20 mites), moderate (21–50 mites), or heavy (> 50 mites).

## Results

**Fig. 2:**
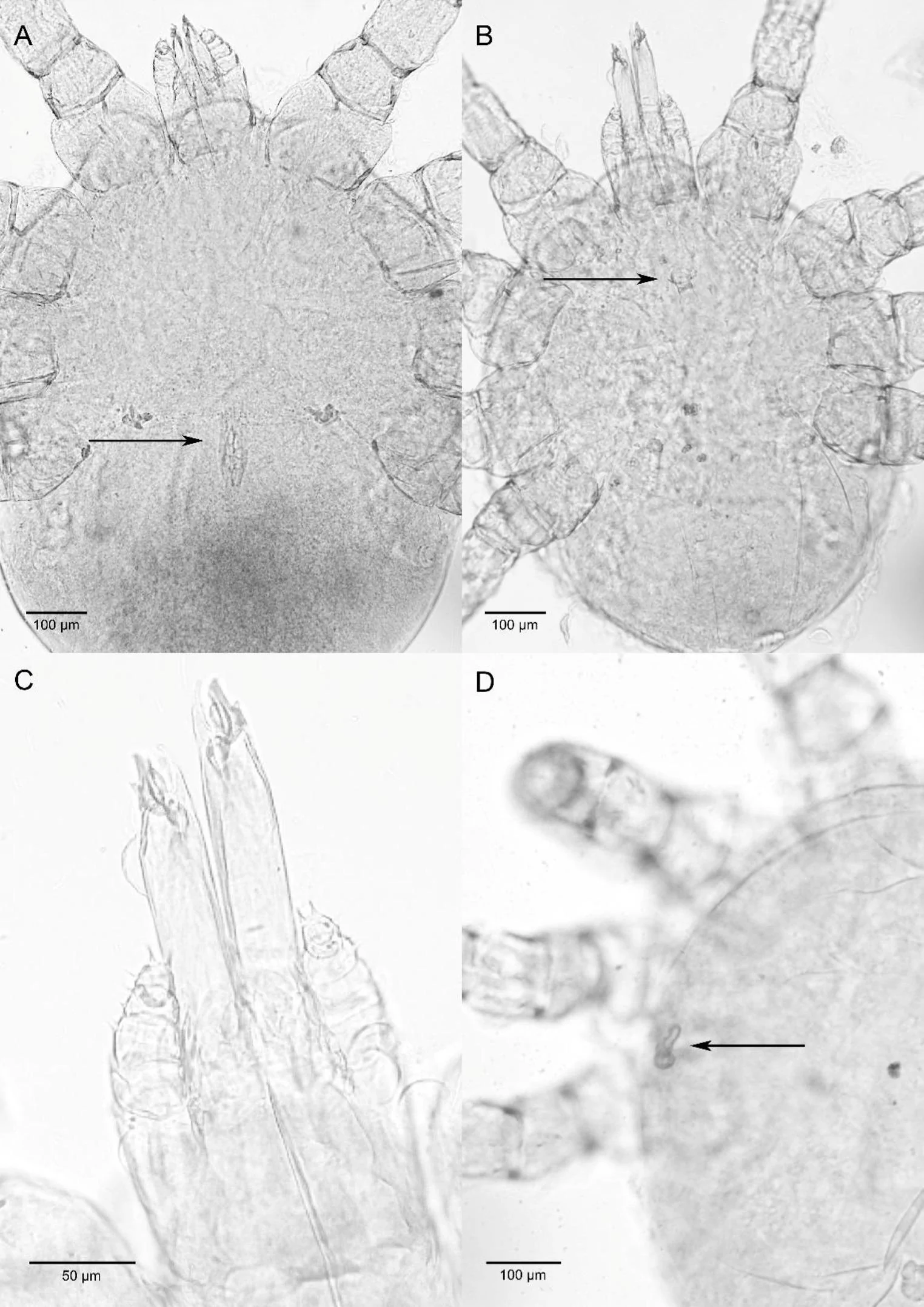
*Larinyssus orbicularis* parasite of Kelp Gulls (*Larus dominicanus*) under light microscopy. **A** Ventral view of female. The arrow indicates the genital plate. **B** Ventral view of male. The arrow indicates the genital pore. **C** Ventral view of the gnathosoma and chelicerae. **D** Stigma with peritreme, dorsolaterally located between legs III and IV

**Fig. 3:**
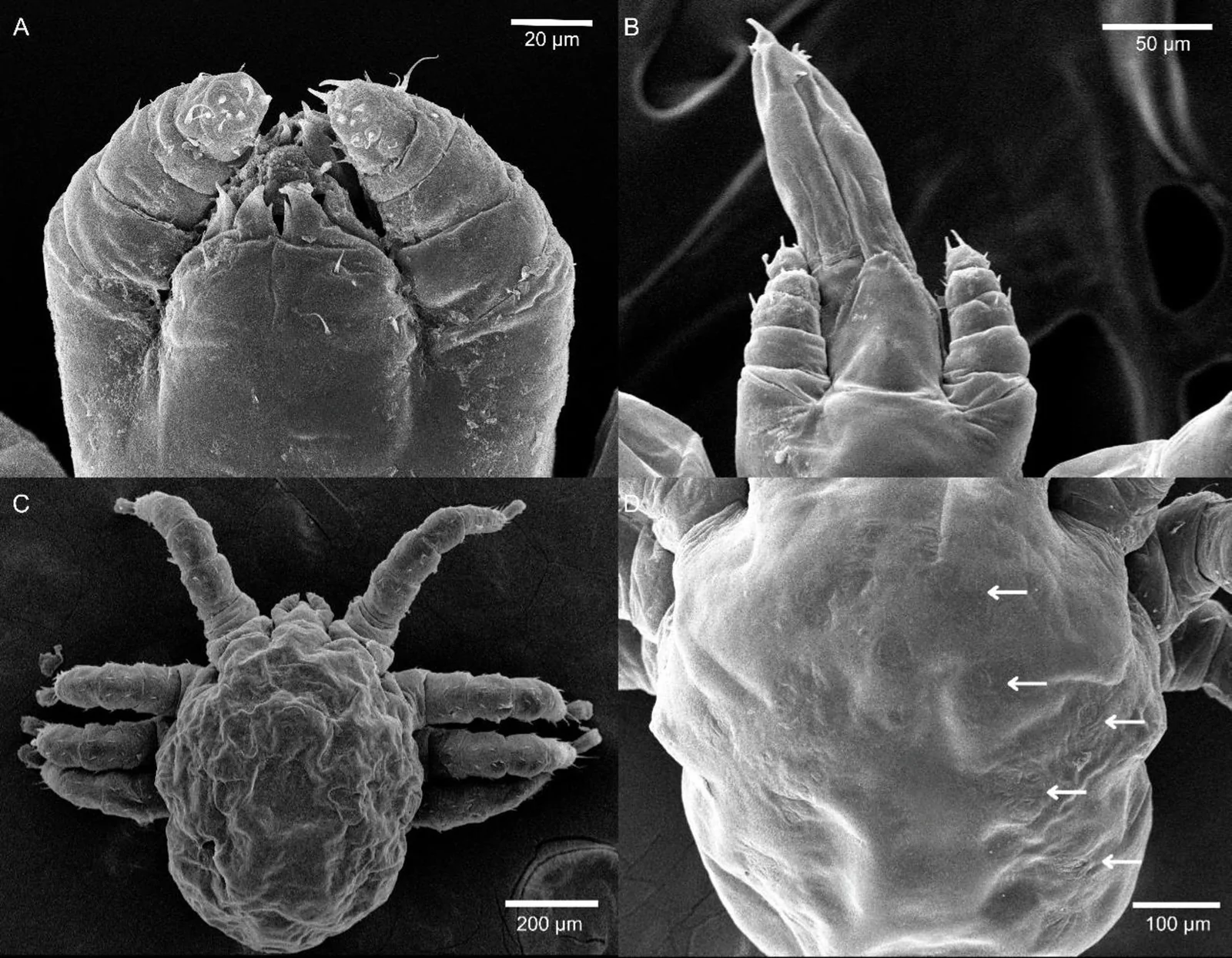
Scanning electron microscopy (SEM) of a female *Larinyssus orbicularis.* **A** Ventral view of gnathosoma. **B** Dorsal view of the gnathosoma with exposed chelicerae. **C** Dorsal view. **D** Detail of the dorsal platelets, a diagnostic characteristic of the species

Fifty-six birds (32.7%) were parasitized by *Larinyssus* mites (Figs. 2 and 3). The complete sample consisted of 2,224 mites, and all specimens of the analyzed subsample were identified as *Larinyssus orbicularis*. Due to the difficulty in visualizing the dorsal plates, which are diagnostic characteristics of the species, under light microscopy, these structures could nevertheless be readily observed using scanning electron microscopy (Fig. 3). Additional characters, including the shape, length, and width of the idiosoma, leg length, chaetotaxy of the dorsum, venter, and gnathosoma, as well as the predominance of longitudinal striations, were consistent with the description of *L. orbicularis* provided by Strandtmann [11].

Mean abundance was 13.0 and the mean intensity was 39.7 mites/host. *Larinyssus orbicularis* was found in 44% (11/25) of the hosts collected from beach surveys and in 30.8% (45/146) of the birds who died during rehabilitation. Of the parasitized hosts, 32 gulls were females, 23 were males, and one individual of indeterminate gender, while 39 consisted of adults, 13 immatures and only 4 juveniles.

The prevalence of nasal mites did not differ between males and females (regardless age class) (*χ*^2^ = 1.34; *p* = 0.248), while it varied among age classes (regardless of gull sex) (*χ*^2^ = 8.99; *p* = 0.011). Pairwise comparisons showed that juveniles had a significantly lower prevalence (11.8%) than immature (43.3%; *χ*^2^ = 8.14; *p* = 0.004) and adult individuals (36.4%; *χ*^2^ = 7.41; *p* = 0.006), whereas no significant difference was found between immatures and adults (*χ*^2^ = 0.47; *p* = 0.492). No significant differences were observed between male and female hosts regarding mean intensity (*U* = 341.0, Z = 0.46, *p* = 0.645) or mean abundance (*U* = 2967.0, Z = 1.03, *p* = 0.300). Similarly, mite infection did not differ significantly among age classes for mean intensity (*H* = 2.05; *p* = 0.358). For mean abundance, although mean rank values were higher for immatures and adults than for juveniles, Dunn's post hoc test with Bonferroni adjustment revealed no significant pairwise differences (all *p* > 0.017) (Table 1).

**Table 1:** Prevalence (P%), mean abundance (MA), mean intensity (MI) and range (R) of *Larinyssus orbicularis* (Rhinonyssidae) in male, female, adult, immature and juvenile Kelp Gull (*Larus dominicanus*) (*n* = 171) collected in Brazil

	P%	MA	MI	R
Male (*n* = 78)	29.5	10.6	35.8	1–155
Female (*n* = 84)	38.1	16.3	42.8	1–197
Adult (*n* = 107)	36.4*	15.6**	42.9	1–197
Immature (*n* = 30)	43.3*	16.5**	38.2	1–149
Juvenile (*n* = 34)	11.8	1.6**	13.8	1–49
Total birds (*n* = 171)	32.8	13.0	39.7	1–197

^*^Pearson's chi-square test: *p* = 0.006 (adults vs. juveniles); *p* = 0.004 (immatures vs. juveniles)

^**^Kruskal-Wallis test: *p* = 0.037 (adults vs. immatures vs. juveniles)

The intensity of infection ranged from 1 to 197 mites per host. According to the proposed classification, 48.2%, 30.3%, and 21.4% of infected hosts were assigned to the low, moderate, and heavy categories, respectively.

**Fig. 4:**
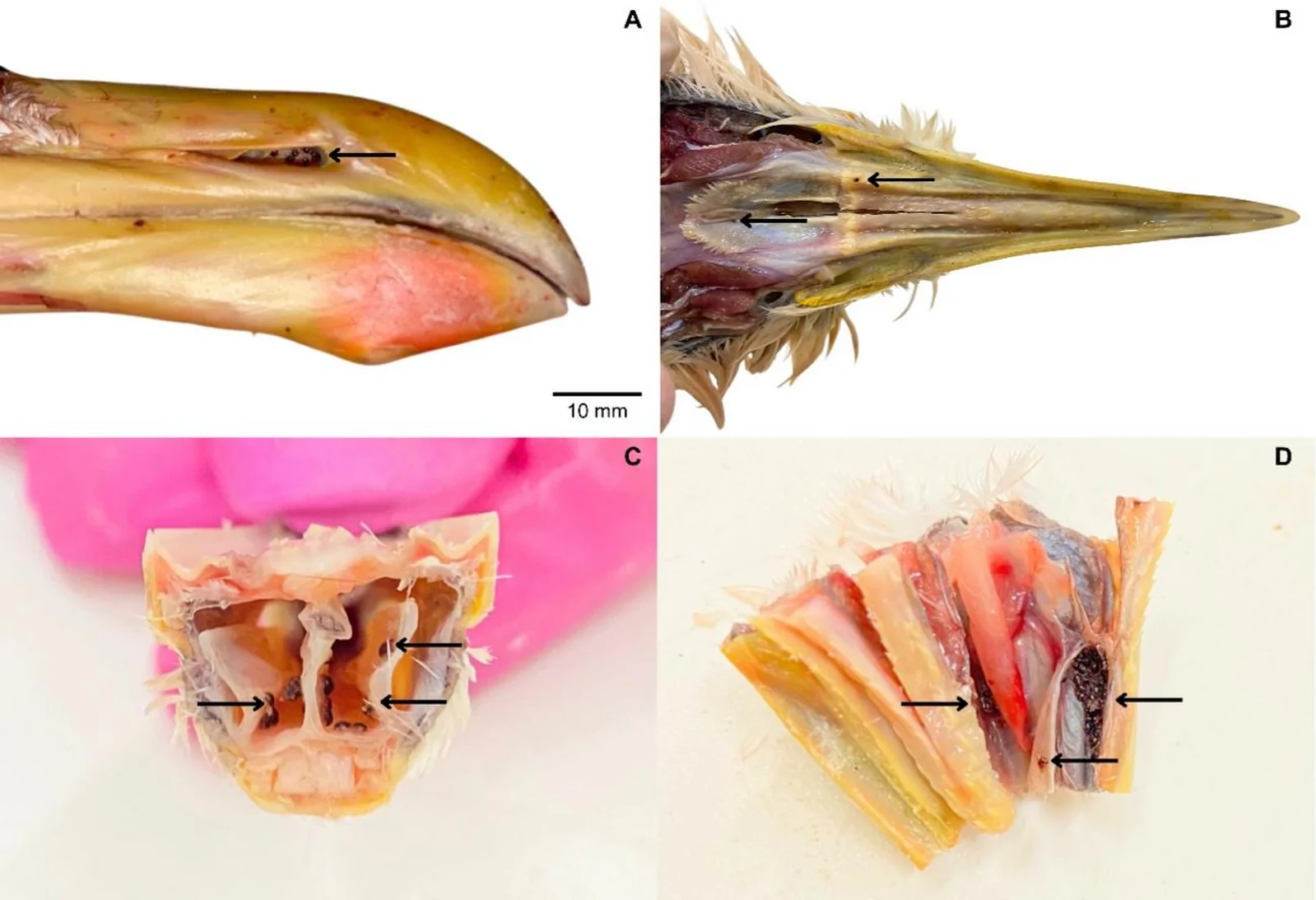
Beak and nasal cavity of Kelp Gulls (*Larus dominicanus*). **A** Mites observed through the nostril. **B** Mites observed in the oral cavity, close to the cleft palate. **C** Mites at rostral nasal conchae. **D** Mites at middle nasal concha

Mites occurred throughout the upper respiratory tract, including the rostral, middle, and caudal nasal conchae. In heavily parasitized birds, these parasites were additionally detected around the nasal-passage opening of the palate (choana) and very close to the nostrils (Fig. 4). On nasal cavity gross pathology, the mites were observed adhered to the hosts mostly with limited amount of mucus, but in some individuals, it was observed some mucoid material accumulated around mites, the presence of large amount of mucus as tenacious mucus and bloody tenacious mucus.

Regarding the origin of the positive hosts, 55 (98.2%) were collected in Santa Catarina and one (1.8%) in Rio Grande do Sul. No mites were found in gulls from the states of São Paulo or Rio de Janeiro (Fig. 1).

## Discussion

Prevalence of Rhinonyssidae mites varies in relation to the host species, with prevalence of 4.5% to 50% recorded in birds associated with aquatic environments [10, 25, 35–39]. *Larinyssus orbicularis* is a widespread species, commonly found in gulls and terns (Laridae) in America, Australia, Africa and Europe. However, there is a lack of information on parasitological indices in most of the recorded hosts (Table 2). The prevalence of *L. orbicularis* in Kelp Gulls in the present study was higher than the indices found in Herring Gulls (*L. argentatus* Pontoppidan, 1763) in the United States (24.8%) and Russia (4.5%) [25, 40]. The elevated parasitological indices observed in this study should also be considered alongside its substantially larger sample size, which exceeded the combined number of birds examined in all previous studies (Table 2). In the United States, 63% (5/8) of adults, 50% (13/26) of immatures and 2% (1/43) of juveniles were parasitized by mites [40]. The data presented here is in line with TerBush [40], with an increase in infection rates in gulls from the second year of life, suggesting that transmission to younger individuals may occur after the chicks have left the nest.

**Table 2:** Review of records of *Larinyssus orbicularis* on a range of host bird species globally and respective references. Prevalence (P%), number of infected hosts (NIH), number of examined hosts (NEH), number of collect mites per host individual (NM). – Information not reported

Host	P% (NIH/NEH)	NM	Locality	Reference
*Anous minutus* Boie, 1844 (White-capped Noddy)	–	–	Australia	Domrow [41]
*Chlidonias leucopterus* (Temminck, 1815) (White-winged Tern)	100 (1/1)	–	Africa	Fain [42, 43]
	100 (2/2)	–	Northern Rhodesia	Fain [44]
	–	–	Russia	Butenko [5]
*Chroicocephalus novaehollandiae* (Stephens, 1826) (Silver Gull)	–	–	Australia	Domrow [41]
*Glareola pratincola* (Linnaeus, 1766) (Collared Pratincole)	100 (1/1)	–	Northern Rhodesia	Fain [44]
*Larus argentatus* Pontoppidan, 1763 (Herring Gull)	11.1 (1/9)	55	United States	Strandtmann [11]
	24.8 (19/77)	–	United States	TerBush [40]
	100 (1/1)	130	United States	Pence [45]
	4.5 (1/22)	–	Russia	De Rojas et al. [25]
*Larus atricilla* Linnaeus, 1758 (Laughing Gull)	20 (1/5)	–	United States	Strandtmann [11]
	100 (1/1)	–	Cuba	Černý & Dusbábek [46]
	100 (2/2)	11/3	United States	Pence [45]
*Larus delawarensis* Ord, 1815 (Ring-billed Gull)	25 (1/4)	–	United States	Strandtmann [11]
	100 (1/1)	1	United States	Pence [45]
*Larus dominicanus* Lichtenstein, 1823 (Kelp Gull)	100 (1/1)	–	Africa	Zumpt & Patterson [47] Zumpt & Till [48]
*Rynchops niger* Linnaeus, 1758 (Black Skimmer)	100 (1/1)	–	Cuba	Černý & Dusbábek [46]
*Sterna hirundinacea* Lesson, 1831 (South American Tern)	9.1 (1/11)	1	Brazil	Silva et al. [36]
*Sterna hirundo* Linnaeus, 1758 (Common Tern)	100 (1/1)	16	United States	Pence [45]
*Thalasseus acuflavidus* (Cabot, 1847) (Cabots Tern)	12.5 (1/8)	36	Brazil	Silva et al. [36]
*Thalasseus bergii* (Lichtenstein, 1823) (Greater Crested Tern)	–	–	Australia	Domrow [49–50]
*Thalasseus maximus* (Boddaert, 1783) (Royal Tern)	37.5 (3/8)	–	United States	Strandtmann [11]

Similar to Rosy-billed Pochard [*Netta peposaca* (Vieillot, 1816)] and Magellanic Penguin [*Spheniscus magellanicus* (Forster, 1781)], our results showed no significant differences in prevalence, mean intensity and mean abundance of infection by Rhinonyssidae between male and female hosts [39, 51]. This could suggest that if transmission occurs during parental care, it is carried out by both parents, since in monogamous birds both males and females take part in chick rearing [22]. Furthermore, if infection occurs after the birds leave the nest, as suggested above, transmission is carried out equally by males and females.

*Larinyssus orbicularis* was first recorded as a parasite of Kelp Gulls in South Africa more than 70 years ago [47, 48]. In Brazil, this species of mite was recorded by Silva et al. [36] parasitizing the South American Tern (*Sterna hirundinacea* Lesson, 1831) and the Cabot's Tern [*Thalasseus acuflavidus* (Cabot, 1847)]. In this previous study, the prevalences of *L. orbicularis* were 9.1% (1/11) for South American Tern and 50% (4/8) for Cabot's Tern*.* Compared to results presented here, these indexes suggest that the prevalence of the same species of mite can differ substantially according to host species. These findings agree with Spicer [52], who suggested that the prevalence of infection is significantly dependent on the bird taxa examined, which indicates that the evolution of the hosts has affected the nasal mite ability to parasitize their avian hosts.

In a pioneering study, Strandtmann [11] reported the occurrence of 55 *L. orbicularis* mites in a single Herring Gull specimen. In the present study, the number of nasal mites found in a single individual reached 197. This is the highest number of *Larinyssus* mites found on a single bird. Proposing a classification based on the patterns observed in this study could serve as an initial framework for future research on infection rates in gulls, and potentially in other birds.

Parasites play a crucial role in ecosystems by influencing host behavior, population dynamics and interactions with other species [53–55]. In this context, classifying the intensity of infection can facilitate the biological interpretation of the observed parasite load, allowing for comparisons between individuals and populations in terms of the severity of parasitism. In addition, it enables the assessment of potential associations between infection intensity and ecological or physiological parameters of hosts. However, because infection rates of nasal mites vary depending on the host species, the categorization proposed here was based on the intensity of infection observed in Kelp Gulls only and should be applied with caution to other bird species. According to Betts et al. [56] different parasite loads prompt different evolutionary dynamics and profoundly shape host evolution by different mechanisms. The high parasite loads observed in this study may suggest that Kelp Gulls are adapted to the presence of *Larinyssus* mites along the upper respiratory tract.

Generally, rhinonyssids do not cause significant harm to the host, but can cause pulmonary congestion, rhinitis, sinusitis and irritation to the nasal epithelium [25, 26]. *Sternostoma tracheacolum* Lawrence, 1948 can invade the lungs of some bird species, causing bronchial dilation and even death of birds [57, 58]. Vanstreels et al. [37] reported reddened nasal mucosa in African Penguins [*Spheniscus demersus* (Linnaeus, 1758)] parasitized by nasal mites and suggested that these parasites can cause mild to moderate sinusitis. Other effects associated with nasal mites in birds are irritation, anemia, or transmission of pathogenic organisms [59]. In terms of pathology, the most detailed research on Rhinonyssidae was carried out by Bell [60] with *S. tracheacolum* in Gouldian Finches [*Chloebia gouldiae* (Gould, 1844)]. The investigation involved studies of both naturally infected wild birds and experimentally infected captive reared birds. On nasal cavity gross pathology, the presence of clear tenacious mucus, bloody mucus and bloody tenacious mucus was observed in parasitized individuals. Even though *S. tracheacolum* is distributed throughout the respiratory tract of the Gouldian Finches and occurs in lower abundance in the nasal cavity, these findings are similar to those found in Kelp Gulls examined in this study.

Trachea, lungs and air sacs histopathological analyzes carried out in passerines infected by *S. tracheacolum* were characterized by a chronic inflammatory response in the lungs associated with mites, as well as parasite fragments [58, 61]. Also based on histopathological analyzes, congested blood vessels in the parenchyma of primary bronchi parasitized, had been reported [27]. Given the predominance of *L. orbicularis* in the nasal cavity, future studies should incorporate histopathological analyses of nasal tissues to determine whether infection by these mites is associated with tissue lesions or other pathological changes in the host.

Even though most of the positive hosts were from the state of Santa Catarina, the low prevalence found in Rio Grande do Sul and the absence of positive hosts in more northern states of Rio de Janeiro and São Paulo may be related to limited sampling sizes. However, prevalence may also be associated with the distribution of host species. In Brazil, Kelp Gulls breed on islands along approximately 850 km of coastline, from Laguna, Santa Catarina, to Rio de Janeiro (Fig. 1) [22]. The species primarily uses the coastal zone for foraging and resting, relying on coastal islands for breeding, and its daily movements are related to food availability, oceanographic conditions, and roosting sites [62]. Both in the coastal zone and in breeding areas, Kelp Gull is observed in groups, including mixed flocks with other species parasitized by *Larinyssus* mites [63].

Rhinonyssids usually disperse by direct transmission from one host individual to another via the oral route, when infected adult birds regurgitate food to their nestlings, or during courtship behavior [3]. Indirect transmission has been observed across water, perches, or other contaminated surfaces and variations in parasite infection rates may reflect natural fluctuations associated with changes in host population density [4]. Fain and Hyland [64] suggested that captive Canaries (*Serinus canarius*), may not be natural hosts for *S. tracheacolum*, but that they have become secondarily infected in areas where they have been introduced as aviary birds. Similarly, *L. orbicularis*, naturally associated with Kelp Gulls, may occasionally infect terns secondarily, as these birds frequently share the same coastal environments in mixed flocks, creating opportunities for interspecific transmission. Thus, areas with high host densities and overlapping species distributions may promote mite transmission among conspecific and heterospecific hosts, contributing to variation in parasite prevalence and infection intensity.

## Conclusion

Among the species of nasal mites, only *L. orbicularis* was found parasitizing Kelp Gulls in southern Brazil. Scanning electron microscopy proved to be a valuable tool for the identification of the mite specimens, providing detailed morphological information that facilitated the observation of diagnostic characters at the species level. Prevalence did not differ between males and females, however, it was significantly lower in juveniles than in immature and adult individuals, corroborating findings from previous studies on gull species, which suggests infections after the nesting period. A high infection intensity was recorded in a single gull, which harbored 197 mites, exceeding all previous records for *Larinyssus* mites. In addition, 21.5% of infected hosts presented heavy infections. Parasitized individuals were found only in the two states located in southern Brazil among the four states surveyed, with most infections recorded in Santa Catarina.

Our results highlight the need for further investigations involving broader geographic sampling across different regions to improve the understanding of the distribution of nasal mites in gulls. In addition, histopathological studies of the nasal cavities are needed to assess the potential effects of these parasites on host tissues and to determine whether they may be associated with respiratory diseases. Observations of clinical signs during the rehabilitation process, together with postmortem examinations of birds that die under rehabilitation, may provide valuable information on host-parasite interactions and the pathological significance of nasal mite infections. Although nasal mite infections cannot yet be readily diagnosed in live birds, individuals that die during rehabilitation represent an important source of information for understanding host-parasite interactions.

## Data Availability

All data supporting the findings of this study are available within the paper. Raw data for each individual is available upon request.
